# Extracellular Polymeric Substances Facilitate the Adsorption and Migration of Cu^2+^ and Cd^2+^ in Saturated Porous Media

**DOI:** 10.3390/biom11111715

**Published:** 2021-11-17

**Authors:** Yuhui Wu, Zhengyu Li, Yuesuo Yang, Diane Purchase, Ying Lu, Zhenxue Dai

**Affiliations:** 1 Key Laboratory of Groundwater Resources and Environment, Jilin University, Ministry of Education, Changchun 130021, China; yuhui18@mails.jlu.edu.cn (Y.W.); luying.819@163.com (Y.L.); dzx@jlu.edu.cn (Z.D.); 2 Jilin Provincial Key Laboratory of Water Resources and Environment, Jilin University, Changchun 130021, China; 3 Academy of Environmental Planning & Design, Co., Ltd., Nanjing University, Nanjing 210093, China; lzy18816215699@163.com; 4 Department of Natural Sciences, Faculty of Science and Technology, Middlesex University, The Burroughs, London NW4 4BT, UK; D.Purchase@mdx.ac.uk

**Keywords:** extracellular polymeric substances, porous media, heavy metals, adsorption, simulation, migration

## Abstract

Heavy metal contamination in groundwater is a serious environmental problem. Many microorganisms that survive in subsurface porous media also produce extracellular polymeric substances (EPS), but little is known about the effect of these EPS on the fate and transport of heavy metals in aquifers. In this study, EPS extracted from soil with a steam method were used to study the adsorption behaviors of Cu^2+^ and Cd^2+^, employing quartz sand as a subsurface porous medium. The results showed that EPS had a good adsorption capacity for Cu^2+^ (13.5 mg/g) and Cd^2+^ (14.1 mg/g) that can be viewed using the Temkin and Freundlich models, respectively. At a pH value of 6.5 ± 0.1 and a temperature of 20 °C, EPS showed a greater affinity for Cu^2+^ than for Cd^2+^. The binding force between EPS and quartz sand was weak. The prior saturation of the sand media with EPS solution can significantly promote the migration of the Cu^2+^ and Cd^2+^ in sand columns by 8.8% and 32.1%, respectively. When treating both metals simultaneously, the migration of Cd^2+^ was found to be greater than that of Cu^2+^. This also demonstrated that EPS can promote the co-migration of Cu^2+^ and Cd^2+^ in saturated porous media.

## 1. Introduction

Heavy metal pollution is a serious global environmental problem, particularly for developing countries—e.g., China [1]. In areas around heavy polluting enterprises, mining areas, sewage irrigation areas, centralized disposal sites of solid waste, and industrial parks and wastelands, improperly treated wastewater can be a major source of heavy metal pollution [2,3,4]. Heavy metals (HMs) in soil reduce soil fertility as well as crop yield and quality [5]. They can also be leached into aquifers through rainfall and irrigation and cause groundwater pollution. Polluted surface water and sewage can also enter groundwater by travelling through porous strata or other hydrogeological connective pathways. Groundwater is the main or even the only source of water supply in arid and semi-arid regions, such as most parts of Africa and North China [6,7,8]. Thus, heavy metals in groundwater pose a real threat to human health.

**Figure 1:**
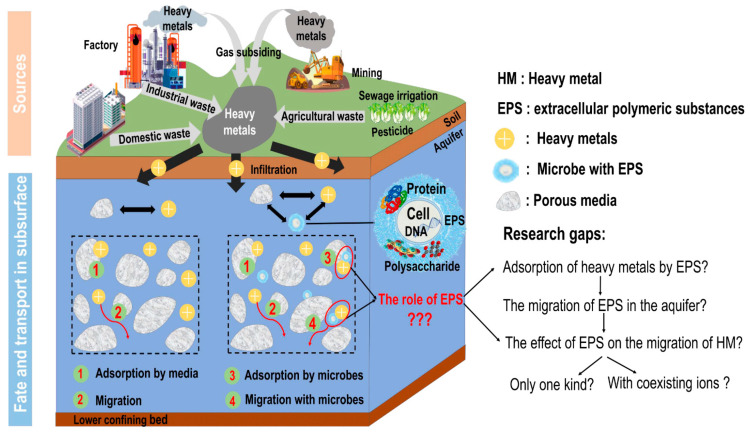
Heavy metals in the subsurface environment: sources, migration, and their interactions with extracellular polymeric substances (EPS).

Some typical sources and migration routes of heavy metals and their potential pathways to subsurface environment are conceptualized in Figure 1. It can be seen that a large number of microorganisms are present in the subsurface environment and that bacteria usually occur as a type of bio-colloid in such an environment [9,10], which poses the question of how they affect the migration of heavy metals in the underground system. Most microorganisms are capable of secreting extracellular polymeric substances (EPS) during their growth [11,12,13]. EPS are polymers of a high molecular weight that are secreted by microorganisms under ambient environmental conditions and they usually include cell lysate and hydrolyzed substances [14]. The main components of EPS are proteins and polysaccharides, as well as nucleic acids, humic acids, and lipids [15]. However, the specific proportion of EPS components is closely related to the types of microorganisms present, the microbial culture conditions, and the EPS extraction methods used. Liu et al. [16] isolated three indigenous DIRB strains, including *Shewanella putrefaciens IAR (iron and As reducing)-S1*, *Shewanella xiamenensis IR (iron reducing)-S2*, and *Klebsiella oxytoca IR-ZA* from the natural groundwater samples of two tube wells of Hangjinhouqi County, China. In addition, bacteria EPS are made up of protein, polysaccharide, organic acid, and a small amount of DNA. Xia et al. [17] found that the main bacteria that may cause clogging in aquifer recharge were *Methylobacterium*, *Janthinobacterium*, *Yersinia*, *Staphylococcus*, and *Acidovorax*. The main components of their EPS are proteins and polysaccharides.

The typical functional groups of EPS usually comprise hydroxyl groups, carboxyl groups, and phenols that can bind to heavy metal ions [18,19,20,21,22,23]. Chen et al. [24] showed that the removal of more than 90% of the heavy metals in biofilm could be attributed to EPS. A series of subsequent studies, as shown in Table 1, indicated that EPS had a strong adsorption capacity for heavy metals.

As revealed by the literature reviews shown above, the effects of EPS on heavy metals in porous media are mainly reflected in two aspects. On the one hand, EPS possess strong adsorption capacity in soil [20]. In aqueous media, microorganisms and EPS will adhere to the surface of sand grains, increasing the friction between the media and fluid. On the other hand, the functional groups on EPS can strongly adsorb heavy metals. Therefore, a large number of extracellular conjugates secreted by microorganisms in aquifers will inevitably affect the adsorption and migration of heavy metals in porous media, leading to changes in the environmental risk of heavy metal pollution.

However, most studies on the interaction between EPS and heavy metals focus on activated sludge, and the research on EPS in groundwater environment mainly focuses on bioclogging in the recharge system [17,29]. Little attention has been paid to the interaction of EPS and heavy metals in aquifers. There are extensive microorganisms in subsurface environments, and how the EPS excreted affect the migration of heavy metals in aquifers is a question that is worthy of further study. In this experiment, the EPS extracted from soil were used to study the effects of EPS to migrate Cu^2+^ and Cd^2+^ in a saturated quartz sand column. This study provides much needed knowledge on the interaction between EPS and heavy metals in porous media, forming a theoretical basis for the migration of heavy metals in aquifers under the influence of EPS. At the same time, this also shows that it is feasible to apply EPS to the removal of heavy metals in porous media in aquifers.

## 2. Materials and Methods

### 2.1. Preparation of Porous Materials

Quartz sand (Sinopharm Chemical Reagent Co., Ltd., Shanghai, China) was selected as a porous medium due to its steady chemical properties. Quartz sand was washed with 0.25 mol/L of hydrochloric acid to remove any surface impurities prior to the experiment, followed by thoroughly rinsing with ultrapure water. The particle size adopted was 0.35–0.4 mm. The cleaned quartz sand underwent autoclaved sterilization (120 °C, 1 h) three times and was then stored in a sterile container at 4 °C under dark conditions for later use [30].

### 2.2. Extraction and Characterization of EPS

The soil used for the EPS extraction was collected at the Chaoyang Experimental Field of Jilin University (Changchun, northeast of China), which has been utilized as a groundwater observation point for a long time. The characteristics of the soil used mimic those of aquifers in the area. We added phosphate buffered saline (PBS) to the soil samples then stirred and shook them. Afterwards, EPS were extracted from the samples through the steam method [31]. A total of 10 mL of the samples was steamed (80 °C, 100 Kpa, 10 min) and then centrifuged while still hot at 8000× *g* for 10 min. During centrifugation, the temperature was reduced to 15 °C. Then, the crude EPS were placed in a dialysis bag of 8000–14,000 Dalton along with deionized water to remove the small molecular substances. The deionized water was replaced every hour. The experimental EPS were obtained after 6 h of dialysis and stored at −20 °C [32].

The levels of EPS protein and polysaccharide were determined by the improved Folin phenol method [33] and the anthrone colorimetric method [34], respectively. The concentration of DNA was determined by the diphenylamine method [35]. The total amount of EPS obtained was expressed as the sum of the polysaccharide and protein concentrations.

Scanning electron microscopy (SEM) and energy-dispersive X-ray spectroscopy (EDS) were used to characterize and analyze changes in the quartz sand surface morphology before and after the experiment.

### 2.3. Procedure of Batch Experiments for Adsorption between EPS, Quartz Sand, and HMs

In order to determine the interaction between EPS and quartz sand, adsorption experiments on different concentrations of EPS in quartz sand were carried out, and 5 g of quartz sand and 30 mL of EPS solution (102.3 mg/L, 136.4 mg/L, 170.4 mg/L, 210 mg/L) were added to each conical flask. Then, the mixture was oscillated (20 °C, 120 rpm, 7 h) and centrifuged (4 °C, 7607× *g*, 15 min). After that, the supernatant was filtered and the EPS concentration was determined. Next, adsorption experiments for heavy metals were carried out on the EPS. A total of 30 mL of the mixed solution of EPS (100 mg/L) and different concentrations of metal ions were added to a sterilized conical bottle. The concentration ranges for Cu^2+^ and Cd^2+^ were 5–40 mg/L and 10–80 mg/L, respectively. The pH of the mixed solution was adjusted to 6.5 ± 0.1 using NaOH or HNO_3_. The conical bottle was sealed and incubated for a period time at 20 °C at 120 rpm. The experimental parameters used for the adsorption of heavy metals on EPS are given in Table 2. Among them, the effect of coexisting ions on the adsorption of target metal ions was studied in groups 5, 6, and 7 (Table 2). The samples were centrifuged as described above and the concentration of heavy metal ions in the filtered supernatant was determined as described in Section 2.6.

EPS have a strong ability to adsorb metals, so the presence of EPS will definitely affect the adsorption of heavy metals in quartz sand. Therefore, adsorption experiments on heavy metals in quartz sand both with and without the presence of EPS were designed to reflect the effect of EPS on the batch adsorption of sand and heavy metals. Sterilized quartz sand (3 g) was added to 30 mL of EPS and a metal ion mixture solution. The EPS concentration of each bottle was 100 mg/L; the concentration of Cu^2+^ was 10, 20, 40, and 80 mg/L; and the concentration of Cd^2+^ was 10, 20, 30, 50, and 80 mg/L. The conical flask was sealed and placed in a shaker, followed by shaking at 20 °C and 120 rpm for 4 h. Then, the supernatant was filtered and the concentration of metal was measured. In the group without EPS, sterilized ultrapure water was added instead of EPS.

### 2.4. Apparatus and Setup for EPS/HMs Breakthroughs

**Figure 2:**
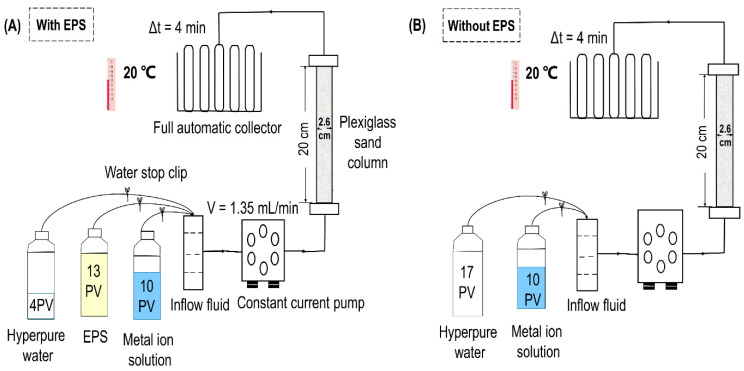
Schematic diagram of experimental device for the EPS/HMs breakthrough: (**A**) HMs breakthrough with EPS; (**B**) HMs breakthrough without EPS. PV—Pore volume; V—Velocity of flow.

The schematic of the experiment set up is presented in Figure 2. The porosity in the column was 0.39 ± 0.01 and the pore volume (PV) was 39.5 ± 1 mL. The indoor temperature was maintained at 20 °C throughout the experiment. The constant-current pump was adjusted to a flow rate of 1.35 mL/min to simulate the groundwater flow rate. The automatic sampler replaced the sampling tube every 4 min and collected the sample once. In order to reduce the effect of gravity on the experiment, the influent entered from the bottom.

### 2.5. Breakthrough of EPS/HMs in Porous Media

EPS inevitably affect the migration of heavy metals in porous media. Therefore, migration experiments with and without EPS were conducted. Contaminated water often contains more than one heavy metal [3]; thus, co-transport experiments on Cu^2+^ and Cd^2+^ were also conducted (Figure 2 and Table 3). Other environmental conditions in groundwater can also affect metal ion migration in the field. Previous researchers have carried out extensive research on the influence of these factors, such as pH, temperature, ionic strength, etc., on the migration of heavy metals in porous media [36,37,38]. On the other hand, as mentioned above, the current interaction between EPS and heavy metals is concentrated in activated sludge. However, there are very few studies on the effect of EPS on the migration of heavy metals in porous media. Therefore, this study focused on the role of EPS and no further study was carried out on other factors. The migration operation conditions of each column are listed in Table 3.

The first set of migration experiment parameters given in Table 3 is the experimental parameters of EPS migration in the sand column. During the experiment, the EPS concentration in the collected samples was measured.

The pH value of the heavy metal ion solution used in the experiment was adjusted to 6.5 ± 0.1 by NaOH or HNO_3_. As shown in Table 3, groups 2, 3, and 4 were experimental groups showing the breakthrough of EPS and metal ions in the column; meanwhile, those with * were the controls showing the breakthrough of metal ions in the column under the corresponding conditions. In the control groups, sterilized ultrapure water replaced the EPS solution. The concentrations of Cu^2+^, Cd^2+^, and EPS of the samples were determined by chemical analysis, as detailed below. The sample of quartz sand taken from the bottom of every sand column after the experiment was air-dried in a centrifugal tube on a super-clean table. SEM and EDS analyses of sand were performed for characterization.

### 2.6. Determination of Cu^2+^ and Cd^2+^ Concentration

All aqueous samples collected were filtered using 0.45 μm membrane filters prior to analysis. The residual concentration of metal ions was then measured by an atomic absorption spectrophotometer (Shimadzu AA-6300C, Shimadzu, Kyoto, Japan) (Cu/Cd: flame; wavelength: 324.8 nm/228.8 nm; lamp cur-rent: 6 mA/6 mA; slit width: 0.7 nm/0.7 nm).

## 3. Results and Discussion

### 3.1. Analysis of EPS Composition

#### 3.1.1. Effect of Dialysis on the Content of EPS Composition

**Figure 3:**
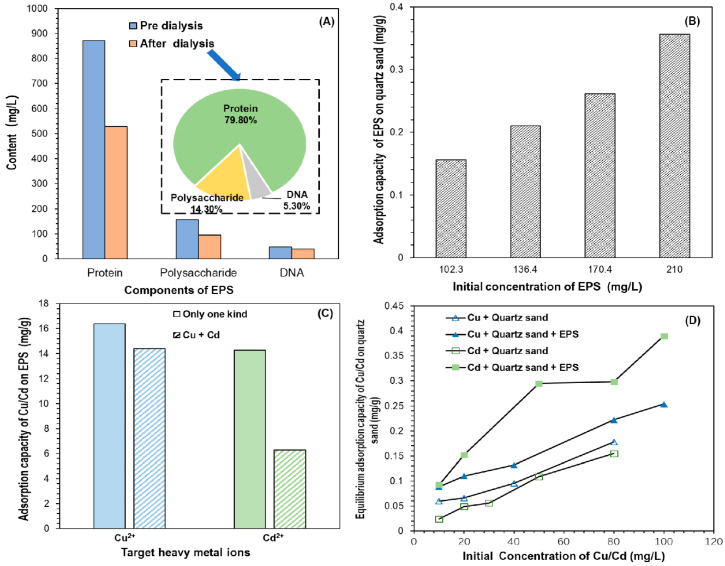
(**A**) Effect of dialysis on the content of EPS composition and the percentage of components of EPS after dialysis, (**B**) experimental results of adsorption (adsorption of different concentrations of EPS on quartz sand), (**C**) effect of coexisting ions on the adsorption of heavy metals by EPS, and (**D**) the influence of EPS on the adsorption of quartz sand with Cu^2+^ and Cd^2+^.

Small molecular substances (below 8000 kDa) contained in crude EPS were extracted by the steam method, and the contents of the EPS composition were found to have changed both before and after dialysis. Among them, the protein, polysaccharide, and DNA contents decreased by 39.3%, 39.2%, and 16.2%, respectively (Figure 3A). This indicated that in the crude EPS, proteins and polysaccharides contained more small molecules. High-molecular-weight adsorbents contain more binding sites and have stronger Van der Waals forces than small molecular substances [39]. Therefore, when extracting EPS from soil using the steam method, a dialysis device should be used for further dialysis and to remove the small molecular substances to reduce the interference in the subsequent experimental results.

#### 3.1.2. Determination of the EPS Composition

After steam filtration and dialysis, EPS were found to be around 79.8% protein and 14.3% polysaccharide, as well as containing a small amount of DNA (5.3%) (Figure 3A). The protein content was the highest, which was consistent with the observation of a number of researchers concerning EPS extracted from aerobic activated sludge [40,41]. The DNA ratio was within the normal range of DNA content (2–15%) proposed by Liao et al. [42], indicating that there were fewer intracellular dissolved substances and that less damage occurred to cells during the extraction process. Therefore, the extraction of EPS by the steam method was effective in this experiment.

### 3.2. Adsorption of EPS on Quartz Sand

Quartz sand has a certain adsorption on EPS, and the adsorption capacity of quartz sand increased with the initial concentration of EPS in the range of 102.3 mg/L–210 mg/L (Figure 3B). However, the total adsorption amount was only 0.356 mg/g when the initial EPS concentration reached 210 mg/L, indicating that the binding force between EPS and quartz sand was weak. This may be related to the fact that the surface of EPS is negatively charged [39] while the surface of quartz sand is generally negatively charged, as well as the fact that the electrostatic attraction between EPS and quartz sand is relatively weak.

### 3.3. Adsorption of Cu^2+^/Cd^2+^ by EPS

#### 3.3.1. Adsorption of EPS with Single Metal Ion

Adsorption assays were carried out at 20 °C, and the adsorption capacity for EPS on Cu^2+^ increased with an increase in the initial Cu^2+^ concentration (5–40 mg/L), which was consistent with the results of Zhang et al. [43]. On the other hand, adsorption first increased with the initial concentration of Cu^2+^, reaching a maximum of 52.5% when the concentration of Cu^2+^ reached 15 mg/L, and then gradually decreased with the increase in the concentration of Cu^2+^. Three isotherm adsorption models were used in the data analysis of the experimental results (Table 4), and the biosorption isotherms of copper followed a typical Temkin behavior. The Freundlich constants n > 1 (Table 4) showed that the adsorption between EPS and copper occurred easily. Similar results have been reported for the adsorption between EPS and Cu^2+^ at 15, 25, 35, and 45 °C, showing that the adsorption results can be well described by the Langmuir model and the Freundlich model [43].

The adsorption trend of Cd^2+^ by EPS was basically the same as that of Cu^2+^ and the adsorption rate reached a maximum of 19.6% when the concentration of Cd^2+^ was 50 mg/L. The Freundlich isothermal adsorption model was able to describe the adsorption process of EPS on cadmium better (Table 4). The coefficient n was less than 1, indicating that the adsorption between EPS and Cd was occurred with relative difficulty.

The adsorption coefficient of the chemical adsorption between the divalent metal ions and the adsorbent can be obtained by the quasi-second-order kinetic adsorption model [44]. In order to predict the adsorption coefficient in this experiment, the adsorption of EPS and Cu^2+^/Cd^2+^ was fitted by the quasi-second-order kinetic model; the fitting parameters are shown in Table 4. The maximum adsorption capacities calculated theoretically by the quasi-second-order kinetic models of Cu^2+^ and Cd^2+^ were 13.46 mg/g and 14.06 mg/g, respectively, which was highly consistent with the actual adsorption capacity. Therefore, the pseudo-second-order kinetic model was able to better simulate the adsorption process of Cu^2+^ and Cd^2+^ by EPS, indicating that the adsorption was dominated by chemical processes [45]. Ion exchange is the main mechanism of the EPS adsorption of Cu^2+^ and Cd^2+^ [27].

Proteins and humus in EPS are strong ligands of Cu^2+^ and Cu^2+^ that can bind to oxygen atoms on carboxyl groups in EPS [46]. At the same time, the main groups in the EPS that interact with Cd^2+^ are carboxyl groups and phosphate groups [19]. Thus, the components in EPS can significantly affect the adsorption of heavy metals by EPS, whilst the contents of EPS components are dependent on their sources and the extraction methods used [34,40,41]. Compared with the maximum adsorption capacity obtained in previous studies [27,45,47], the maximum adsorption capacity obtained in this study was lower. Yin et al. [27] extracted EPS from *Aspergillus fumigatus* by the cation exchange resin (CER) method. The content of the EPS included proteins (24.4 mg/L), polysaccharides (544.4 mg/L), nucleic acid (25.4 mg/L), and uronic acid (324.3 mg/L). Additionally, the maximum sorption capacities were 40 mg/g EPS for Cu and 85.5 mg/g EPS for Cd. Therefore, using the steam extraction method might have reduced the maximum adsorption capacity of EPS on the two heavy metal ions obtained in this experiment.

#### 3.3.2. Adsorption of Both Heavy Metals by EPS

Considering the presence of more than one metal ion in the environment, the adsorption of EPS on two heavy metal ions was investigated (Figure 3C). Coexisting ions reduced the adsorption of EPS on target metal ions. When Cd^2+^ was present as the coexisting ion, the adsorption capacity for EPS on Cu^2+^ decreased from 16.39 mg/g to 14.38 mg/g, and the adsorption capacity decreased by 12.3%. For Cu^2+^, the adsorption capacity of EPS to Cd^2+^ decreased from 14.27 mg/g to 6.283 mg/g, and the adsorption capacity decreased by 56.0%. By comparison, the presence of Cu^2+^ had a more significant effect on the adsorption capacity of Cd^2+^ by EPS. This indicated that under the experimental pH (6.5), the affinity of EPS for heavy metal ions in the Cu^2+^ + Cd^2+^ system was Cu^2+^ > Cd^2+^. The study by Comte et al. [48] also showed that the number of Cu and Cd binding sites on EPS was Cu > Cd at pH = 6, 7, 8. This may be related to two aspects. On the one hand, the spatial effect of functional groups such as carboxyl, amino, phosphate, and hydroxyl groups can hinder the adsorption of large-sized ions by EPS. EPS mainly relies on chemical groups on the surface to coordinately bind or covalently bond with heavy metal ions, thereby adsorbing heavy metals. The ion radius of Cu^2+^ is smaller than that of Cd^2+^, and the functional groups on the surface of EPS have less barriers, meaning that Cu^2+^ is more easily adsorbed by EPS than Cd^2+^. On the other hand, the charge numbers of Cu^2+^ and Cd^2+^ are the same. However, the hydration ion radius of Cu^2+^ is smaller than that of Cd^2+^ and the charge density of Cu^2+^ is higher than that of Cd^2+^. Therefore, the Cu^2+^ has a stronger affinity with the adsorption site and is more likely to preferentially adsorb to the EPS than Cd^2+^.

### 3.4. Effect of EPS on Adsorption of Cu^2+^/Cd^2+^ on Quartz Sand

Previous experiments have shown that EPS has a certain adsorption capacity for Cu^2+^ and Cd^2+^. When EPS exist in quartz sand, there will be competitive adsorption between EPS and HMs on the sand, thus reducing the adsorption of heavy metals on the surface of quartz sand (Figure 3D). The effect of EPS on the adsorption of heavy metals by quartz sand was different and the effect on Cu^2+^ was smaller than that on Cd^2+^. In the system containing EPS, when the initial concentration of Cu^2+^ was between 10 mg/L and 80 mg/L, the amount of Cu^2+^ adsorbed by quartz sand was reduced by about 0.03–0.04 mg/g. When the initial concentration of Cd^2+^ was between 10 mg/L and 80 mg/L, the amount of Cd^2+^ adsorbed by quartz sand decreased in the range of 0.07–0.18 mg/g and the amount of adsorption varied greatly. This may be related to the different adsorption strengths of quartz sand and EPS on Cu^2+^ and Cd^2+^.

In a mixed system of EPS + heavy metal ions + quartz sand, some of the EPS should be adsorbed on the surface of quartz sand. The EPS adsorbed on the surface of quartz sand will have a certain adsorption of heavy metal ions in water, which may increase the adsorption of heavy metals by quartz sand. However, the adsorption experiments carried out on EPS and quartz sand showed that the bonding force between EPS and quartz sand was weak, meaning that most of the EPS were not adsorbed and existed in a free state, thus producing a greater adsorption of heavy metals. The overall results showed that the adsorption of heavy metal ions by free EPS in water weakened the adsorption of heavy metals by quartz sand.

### 3.5. Breakthrough of EPS in Saturated Porous Media

EPS had a strong migration ability in the quartz sand column. Quartz sand was able to adsorb EPS to a certain extent and retard the migration of EPS in the sand column. The penetration curve mutated at 1.6 PV, then the concentration of EPS in the effluent increased rapidly. After 2.4 PV, the rate of increase slowed down and tended to fluctuate. After 5 PV, the penetration curve tended to be relatively stable and reached the plateau stage. At this time, C/C_0_ was about 0.67.

**Figure 4:**
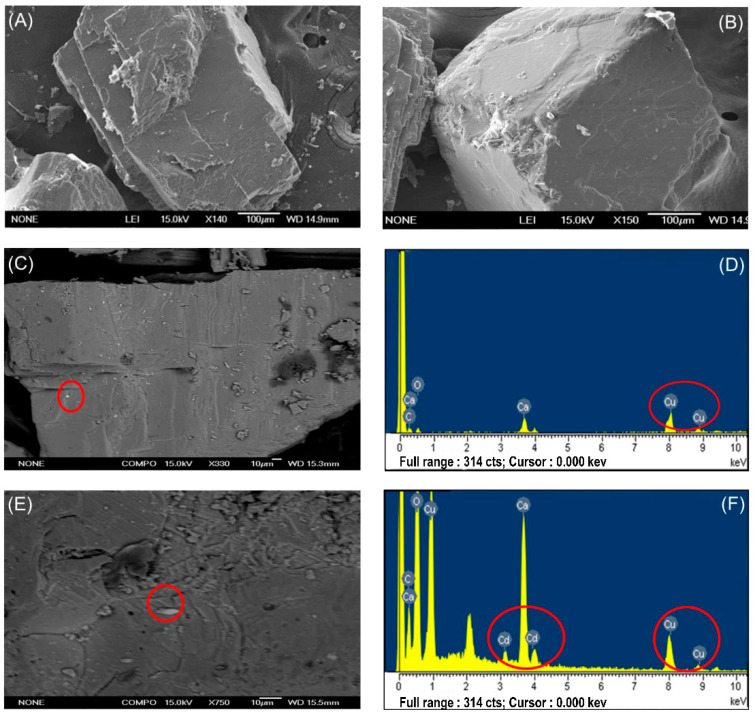
Scanning electron microscopy (SEM) and energy-dispersive X-ray spectroscopy (EDS) spectra of quartz sand under different migration conditions: (**A**) original sample prior to the experiment; (**B**) EPS breakthrough; (**C**,**D**) breakthrough of EPS + Cu and the corresponding EDS spectra; (**E**,**F**) breakthrough of EPS + Cu + Cd and the corresponding EDS spectra.

SEM analyses were carried out on quartz sand samples taken from above columns, and the surface of the quartz sand after the EPS adsorption showed no obvious changes (Figure 4A,B). It was further proven that the binding effect of EPS on quartz sand was weak, and only a small part was adsorbed on the surface of quartz sand, which was consistent with our previous adsorption results.

### 3.6. Effect of EPS on the Cu^2+^/Cd^2+^ Migration through Saturated Porous Media

#### 3.6.1. Effect of EPS on Migration of Individual Metal

**Figure 5:**
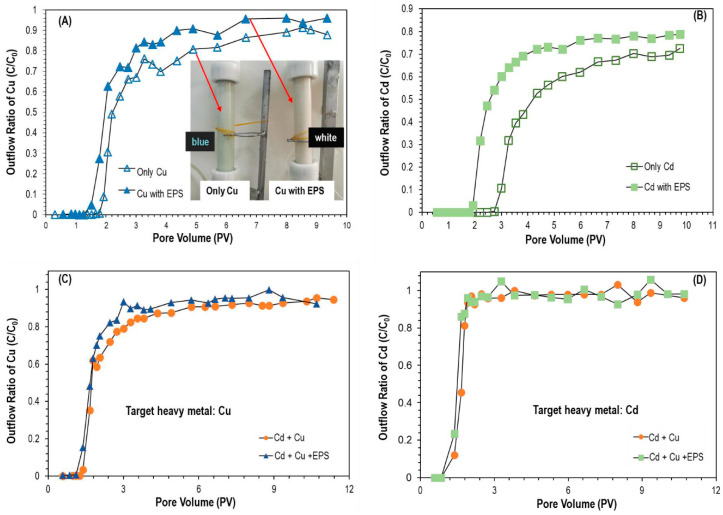
Migration experiments carried out on EPS and metal ions under different conditions: (**A**,**B**) migration of Cu/Cd in the presence or absence of EPS; (**C**,**D**) effect of Cu and Cd co-migration in the presence of EPS.

EPS can significantly promote the migration of Cu^2+^/Cd^2+^ in saturated porous media (Figure 5A,B). When the solution of Cu^2+^ flowed through an EPS-saturated quartz sand column, it penetrated at a pore volume of 1.55 PV (C/C_0_ > 0.01), which was obviously earlier than that without an EPS-saturated sand column (pore volume of 1.7 PV). At the same time, when Cu^2+^ flowed through an EPS-saturated sand column, the C/C_0_ was about 0.95 at the platform stage, which was higher than the value of 0.9 achieved in EPS-free conditions.

**Figure 6:**
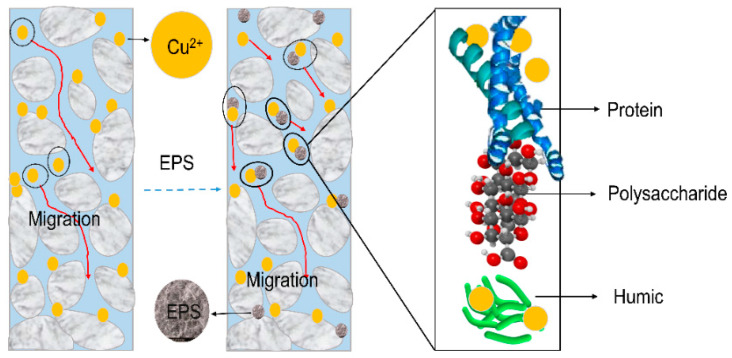
Schematic representation of Cu migration mechanism in saturated porous strata.

It can be seen in the experiment that the pure white quartz sand column without saturated EPS solution appeared a light blue color due to the presence of Cu^2+^ solution (Figure 5A). However, in the quartz sand column saturated with EPS solution, no blue coloration was observed. It was evident that EPS promoted the migration of Cu^2+^ in the quartz sand column and that more Cu^2+^ was released from the sand column compared with the unsaturated group. Therefore, the sand column did not appear a pale blue color. There was still residual Cu^2+^ in the sand column, which could be detected by SEM and EDS analysis results (Figure 4C,D). In the SEM characterization, bright spots of copper appeared on the surface of the quartz sand, and there were weak copper peaks in the EDS spectrum. Figure 6 illustrates the migration mechanism of Cu in saturated porous strata and schematically represents the influence of EPS on Cu migration. When Cd^2+^ solution flowed through the EPS-saturated quartz sand column, it penetrated the column at a pore volume of 1.9 PV (C/C_0_ > 0.01), which was 0.9 PV earlier than the penetration time of the column saturated without EPS. After that, the concentration of Cd^2+^ increased rapidly and slowed down at 3.8 PV (without EPS, 5.4 PV), before stabilizing after 6.6 PV (without EPS, 8.0 PV). At the plateau stage, C/C_0_ was about 0.78, higher than the value of 0.7 gained without EPS.

By comparing A and B in Figure 4, it can be seen that EPS showed a greater influence on the migration of Cd^2+^ in porous media than on the migration of Cu^2+^, which was consistent with the results of the batch adsorption experiment.

During the flow of Cu^2+^ and Cd^2+^ through the quartz sand column, the EPS concentration in some effluent was detected. It was found that EPS migrated from the column together with Cu^2+^ and Cd^2+^ in the sand column saturated with EPS, indicating that EPS desorption occurred on the quartz sand. The binding force between EPS and quartz sand was weak and EPS bound to metal ions when positively charged Cu^2+^ and Cd^2+^ penetrated through the quartz sand column. EPS adsorbed competitively with quartz sand, bringing heavy metal ions out of the sand column and promoting the migration of Cu^2+^ and Cd^2+^. As a result, the penetration time of Cu^2+^ and Cd^2+^ in the experiment was significantly advanced when flowing through the sand column after EPS saturation, and the outflow ratio in the plateau period was higher than that in the control experiment. Studies have shown that bacterial colloid is an excellent carrier of Hg, Zn, and Cd, and the migration rate of metal adsorbent is 4 to 6 times that of dissolved metal [49]. Pang et al. [50] reported that bacteria can significantly promote Cd^2+^ migration when Cd^2+^ migrates with bacteria, which increases the migration rate by 17–20 times. As part of the bacterial composition, EPS also play important roles in these migration processes, and the results of this experiment have confirmed this conclusion.

#### 3.6.2. Effect of EPS on Co-Transport of Cu^2+^ and Cd^2+^

When a variety of metal ions migrated in a porous medium, EPS enhanced the migration according to the affinity of EPS and heavy metal ions. The stronger the affinity, the more obvious the promotional effect. From Figure 5C,D, it can be seen that when a mixture of Cu^2+^ and Cd^2+^ flowed through a quartz sand column saturated by EPS, the breakthrough time of Cu^2+^ and Cd^2+^ was advanced by about 0.2 PV and 0.1 PV, respectively. This was due to the EPS desorption from quartz sand and binding to Cu^2+^ and Cd^2+^, but the binding abilities of Cu^2+^ and Cd^2+^ to EPS were different. The previous batch of adsorption results showed that the affinity of EPS was Cu^2+^ > Cd^2+^, meaning that the effect of EPS on the Cu^2+^ penetration curve was slightly larger than that of Cd^2+^, while the promotion of Cu^2+^ migration was slightly stronger than that of Cd^2+^. By comparing the single migration (Figure 5A,B) and the co-migration of Cu and Cd (Figure 5C,D), it could be seen that the migration of Cd^2+^ in the quartz sand column was more sensitive to the influence of coexisting ions than that of Cu^2+^. The presence of Cu^2+^ played a relatively major role in promoting the migration of Cd^2+^ in porous media.

Meanwhile, SEM and EDS (Figure 4E,F) also showed that, after the mixed solution of Cu^2+^ and Cd^2+^ penetrated through the EPS saturated quartz sand column, both Cu^2+^ and Cd^2+^ remained in the sand column. Copper and cadmium highlights appeared on the surface of sand grains and strong copper and cadmium peaks were seen in the EDS spectrum analysis. This indicated that the coexisting ions Cu^2+^ and Cd^2+^ both adsorbed competitively with EPS and migrated with EPS, leaving more of them in the sand column than in the case of single-ion migration, which was consistent with the analysis of the penetration experiment results.

## 4. Conclusions

Due to the fact that a large number of microorganisms exist in soil and aquifers, they must be taken into account in the fate and transport of heavy metals in aquifers. This study demonstrated that EPS can significantly promote the migration of heavy metal ions in saturated porous strata. It was shown that the EPS produced by soil microorganisms affected the physio-chemical properties of heavy metals in aquifers. EPS showed a good adsorption to Cu^2+^ and Cd^2+^ governed by the Temkin and Freundlich models, and we found that the maximum adsorption capacities of EPS for the two metals were 13.46 and 14.06 mg/g, respectively. The bonding strength between EPS and quartz sand was weak, meaning that EPS can significantly promote the migration of the two metals in the sand column. This work also showed that the prior saturation of the porous medium with EPS solution can enhance the removal of heavy metals, provided a new treatment option for aquifers with heavy metal pollution.

## Figures and Tables

**Table 1:** Adsorption of heavy metals (HMs) on extracellular polymeric substances (EPS).

The Sources of Microorganisms	Microorganisms Types	The Kind of Metal	Adsorption Capacity or Adsorption Efficiency	Reference
Wastewater sludge systems	*Klebsiella* sp., *Bacillus* sp.	Hg(II)	2597.62 mg/g (*Klebsiella* sp.), 2617.23 mg/g (*Bacillus* sp.)	[25]
Aqueous environment	*Agrobacterium tumefaciens F2*	Pb^2+^, Cd^2+^, and Ni^2+^	94.67% (Pb^2+^), 94.41% (Cd^2+^), 77.95% (Ni^2+^)	[3]
Wastewater treat plant	*D. desulfuricans (GenBank/HQ022824.1)*	Cu^2+^, Zn^2+^	899.1 mg/g EPS for Cu^2+^, 932.1 mg/g EPS for Zn^2+^	[26]
-	*Aspergillus fumigatus*	Cu(II), Cd(II)	40 mg/g EPS for Cu(II), 85.5 mg/g EPS for Cd(II)	[27]
Activated sludge in municipal wastewater treatment plants	*Klebsiella* sp. *J1*	Pb(II)	99.5 mg/g	[28]

**Table 2:** Parameter setting of EPS and heavy metal adsorption experiment.

No.	Concentration of Cu (mg/L)	Concentration of Cd (mg/L)	Contact Time (min)
1	5	0	5, 15, 30, 50, 70, 100, 120, 240, 360, 720
2	5, 10, 15, 25, 40	0	720
3	0	7	5, 15, 30, 50, 70, 100, 240, 720
4	0	10, 20, 30, 50, 80	720
5	0	5	720
6	0	7	720
7	5	7	720

**Table 3:** Parameter setting of migration experiment.

No.	Hyperpure Water	EPS (PV) (50 mg/L)	Cu^2+^ (PV) (100 mg/L)	Cd^2+^ (PV) (50 mg/L)
1	4	13	-	-
2	4	13	10	-
2 *	17	-	10	-
3	4	13	-	10
3 *	17	-	-	10
4	4	13	5	5
4 *	4	-	5	5

* Corresponding control group. PV—Pore volume.

**Table 4:** Model parameters of the EPS adsorption of Cu^2+^ and Cd^2+^.

	Isothermal Adsorption Model							Pseudo-Second-Order Kinetic Model			
	Freundlich Constants			Linear Model Constants		Temkin Constants					
	K	n	R^2^	K	R^2^	K	R^2^	Q_e,exp_ (mg/g)	K (g/(mg h))	R^2^	Q_e,cal_ (mg/g)
Cu^2+^	14.02	1.409	0.904	3.908	0.883	44.704	0.963	20.79	2.208	0.993	13.46
Cd^2+^	0.372	0.697	0.974	2.327	0.956	66.496	0.894	15.93	−0.568	0.997	14.06
